# Analysis of Depression and Anxiety Scores Following Initiation of Elexacaftor/Tezacaftor/Ivacaftor in Adults With Cystic Fibrosis

**DOI:** 10.1111/crj.70007

**Published:** 2024-08-29

**Authors:** Harish Pudukodu, Margret Z. Powell, Agathe Ceppe, Scott H. Donaldson, Jennifer L. Goralski, Nathaniel A. Sowa

**Affiliations:** ^1^ Department of Psychiatry Brigham and Women's Hospital Boston Massachusetts USA; ^2^ Department of Psychology and Neuroscience Baylor University Waco Texas USA; ^3^ Department of Medicine, Division of Pulmonary Diseases and Critical Care Medicine University of North Carolina‐Chapel Hill School of Medicine Chapel Hill North Carolina USA; ^4^ Department of Psychiatry University of North Carolina‐Chapel Hill School of Medicine Chapel Hill North Carolina USA

**Keywords:** adult, anxiety, cystic fibrosis, depression, elexacaftor/tezacaftor/ivacaftor

## Abstract

**Objective:**

Elexacaftor/tezacaftor/ivacaftor (E/T/I) has provided life‐changing pharmacotherapy for many people with cystic fibrosis (CF), but conflicting literature exists regarding the effect on mental health. While some reports suggest E/T/I may induce adverse psychiatric symptoms, others report improvements in mental health symptoms. To add to this growing body of knowledge, we retrospectively analyzed depression and anxiety symptoms before and after E/T/I initiation in adults with CF at a single large US CF center.

**Method:**

Patient Health Questionnaire‐9 (PHQ‐9) and Generalized Anxiety Disorder‐7 (GAD‐7) scores recorded in a database were studied. Patients with scores collected before and after E/T/I initiation were included. Regression analyses described associations between score changes and age, race, ethnicity, sex, CFTR variant, and prior depression and/or anxiety diagnoses. Secondary analyses examined possible confounding effects of the COVID‐19 pandemic.

**Results:**

There was no change in mean GAD‐7 (0.5 ± 5.3, *p* = 0.41) or PHQ‐9 (−0.02 ± 6.0, *p* = 0.97) scores following initiation of E/T/I (*N* = 86). A trend between a prior diagnosis of depression and worsening in PHQ‐9 post‐E/T/I was observed (OR 3.58; *p* = 0.054).

**Conclusions:**

Treatment with E/T/I does not lead to changes in depression or anxiety symptoms at the population level in this single center cohort study. A prior diagnosis of depression trended towards an increased odds of worsening PHQ‐9 scores after E/T/I initiation.

## Graphical abstract

Treatment with elexacaftor/tezacaftor/ivacaftor does not lead to changes in depression or anxiety symptoms at the population level. There did not appear to be an effect of the COVID‐19 pandemic on these results.

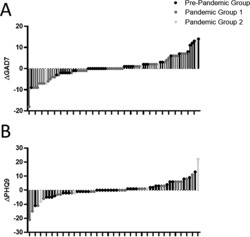

## 1 Introduction

Cystic fibrosis (CF) is a multisystem disorder with significant chronic respiratory and gastrointestinal tract manifestations [1, 2]. People with cystic fibrosis (PwCF) have high comorbid rates of depression (25%) and anxiety symptoms (35%), which are associated with decreased lung function, low BMI, and reduced treatment adherence [3, 4]. In recent years, there has been significant progress made in CF treatment and outcomes with the advent of cystic fibrosis transmembrane conductance regulator (CFTR) modulators [3, 5, 6, 7, 8, 9, 10, 11, 12, 13, 14, 15]. CFTR modulators are targeted therapeutics that restore CFTR protein function [5, 8, 11, 12, 16]. There are currently four CFTR modulators approved for use in the United States, and all have demonstrated significant improvements in, or long‐term stabilization of, lung function, exacerbation frequency, and quality of life in pwCF [3, 5, 6, 7, 9, 11, 16, 17, 18, 19]. Elexacaftor/tezacaftor/ivacaftor (E/T/I) is approved for use in pwCF with at least one copy of the F508del‐CFTR variant or another responsive CFTR gene variant, making ~90% of US CF patients eligible for this highly effective modulator treatment (HEMT) [16, 20].

While CFTR modulators have improved the health of many patients with CF, their effect on mental health symptoms have been less clearly evident [3, 21].There have been several case reports/series describing adverse psychiatric effects following initiation of a CFTR modulator, predominantly in the form of worsening anxiety, depression, and suicidal ideation [22, 23, 24, 25].Prospective longitudinal studies are generally small and show a high degree of variability in improvement, worsening, or stable symptoms; they have generally been conducted outside the United States due to more recent regulatory approvals for E/T/I compared with the United States. A review of real‐world safety of CFTR modulators identified limited evidence of mental health‐related adverse effects with E/T/I [26, 27, 28]. Most recently, a comprehensive review of safety databases, prior clinical trial data, and post‐marketing adverse event reports, in total covering over 60 000 patients with CF, found exposure‐adjusted rates of depression events to be similar between people treated with E/T/I and a pooled placebo group [29]. Notably, this review looked at depression/suicidality and not anxiety or other mental health concerns. Thus, while there is a growing body of literature that suggests E/T/I may not affect the mental health of pwCF at the population level, concerns are still voiced by patients initiating therapy. Challenges remain in predicting which individuals may experience changes in their mental health after CFTR modulator initiation. Moreover, many of these prior studies have failed to account for changes in psychotropic medications or pre‐existing anxiety/depression that may confound outcomes.

Given the widespread use of HEMT, it is imperative to continue to gain a greater understanding of potential psychiatric sequelae. While prospective data are vital to collect, accumulating additional retrospective data is more practical, and potentially the most realistic option currently available in the United States, given that there remain few treatment‐naïve pwCF. In this retrospective study, depression and anxiety symptom scores pre‐ and post‐ E/T/I treatment initiation are compared in an adult CF clinic patient population to systematically interrogate the possibility of significant changes in depression and anxiety symptomatology. We also specifically examine whether a prior history of depression or anxiety is associated with any change in psychiatric symptoms after E/T/I initiation. Some of these data have been previously reported in the form of an abstract [30].

## 2 Materials and Methods

We performed a retrospective analysis of mental health (MH) screening data collected from adults with CF at the University of North Carolina‐Chapel Hill (UNC). The study was approved by the UNC Institutional Review Board. MH screening is conducted at least annually using the Patient Health Questionnaire‐9 (PHQ‐9) [31] and Generalized Anxiety Disorder assessment (GAD‐7) for depression and anxiety, respectively [32]. Clinically obtained MH screening data are entered into a REDCap database [33]. To be included in this analysis, pwCF had to have initiated treatment with E/T/I and have had at least one PHQ‐9 and GAD‐7 score recorded before starting treatment, and at least one PHQ‐9 and GAD‐7 score collected after starting E/T/I. Subjects with less than 14 days between the E/T/I initiation and the follow‐up screeners were excluded from the analysis, as the measurement tools ask for symptoms experienced within “the past 2 weeks.” Deidentified demographic and psychiatric assessment data abstracted from the electronic medical record included CFTR modulator prescription data, prior mental health diagnoses, psychiatric medication prescriptions at time of E/T/I initiation, and CFTR variant data.

Interpretation of PHQ‐9 and GAD‐7 scores best occurs in the context of knowledge of psychotropic medication use. For example, a high score on a mental health screener could result in the addition of a new medication or an increase in dosage. Conversely, a lower score might be entered into the database, but the screener was conducted after a change in medication had already occurred based on clinical symptoms without formal screening assessments completed. For this reason, we extracted data from the electronic health record about psychiatric medication prescriptions during the 6 months pre‐E/T/I period and any medication changes that happened within 6 months post‐E/T/I initiation. Psychiatric medications were defined as any antidepressants, mood stabilizers (defined as lithium, valproic acid, carbamazepine, and lamotrigine) anxiolytics, antipsychotics, or sedative hypnotics, regardless of indication for use. Hydroxyzine was also included when indicated that it was prescribed for anxiety. See Table S1 for full report of medication use pre‐E/T/I. The patients were cohorted into the following groups: (1) added psychiatric medication; (2) decreased dose or stopped medication; (3) increased dose; (4) multiple changes made; (5) no medication changes made; and (6) no psychiatric medications prescribed.

All data analyses were performed with SAS 9.4 (Cary, NC, USA). The intra‐subject change in GAD‐7 and PHQ‐9 total scores were calculated using screening data captured at the closest visit prior to and following E/T/I initiation. A clinically significant change in PHQ‐9 or GAD‐7 score is defined as a change of score ≥5 in either direction (decrease = clinical improvement; increase = clinical worsening) [31]. Comparisons of continuous scores pre‐ to post‐E/T/I initiation were made with paired t‐tests. Associations between continuous changes and predictors were evaluated with linear regressions. Associations between categorical changes (worsening vs not worsening) and predictors were evaluated with logistic regressions; *p* values <0.05 were considered significant.

To evaluate whether the SARS‐CoV‐2 (COVID‐19) pandemic may have confounded results, we performed secondary analyses in three different groups of subjects: (1) E/T/I initiation and all mental health data collected prior to onset of the COVID‐19 pandemic (Pre‐Pandemic Group), (2) baseline PHQ‐9 and GAD‐7 scores collected pre‐pandemic, but post‐initiation scores collected during pandemic (Pandemic Group 1), and (3) all PHQ‐9 and GAD‐7 data collected during the pandemic (Pandemic Group 2). Onset of the COVID‐19 pandemic was defined as March 11, 2020, according to the World Health Organization pandemic declaration [34].

## 3 Results

### 3.1 Baseline Population Description

Eighty‐six pwCF were included in the study; out of these, 81 had GAD‐7 data available before and after E/T/I initiation and 82 had paired PHQ‐9 data. Table 1 provides relevant demographic data. The median [IQR] time between the baseline mental health screening and drug initiation was 403 days [321, 497] days. The median time between E/T/I initiation and mental health screening after E/T/I initiation was 163 [71, 305] days. Thirty‐two patients (36.4%) had elevated GAD‐7 scores (i.e., ≥5), and 35 (39.8%) had elevated PHQ‐9 scores (i.e., ≥5). A higher percentage of women (46%) had elevated GAD‐7 scores at baseline compared with men (23%). Sex was not associated with elevated PHQ‐9 scores at baseline. Race, ethnicity, age, CFTR variant, and the percent of predicted forced expiratory volume in 1 s (FEV_1_pp) were not associated with elevated GAD‐7 or PHQ‐9 scores at baseline. To provide further assurance regarding baseline scores, we also evaluated mean scores between 2016 and 2018 (GAD‐7 = 5.4 ± 6.1; PHQ‐9 = 6.2 ± 6.4), and found no difference between these earlier scores and the pre‐E/T/I scores ultimately used in the final analyses.

**TABLE 1:** Patients initiated on elexacaftor/tezacaftor/ivacaftor.

	*N* (%)
*Sex*	
Female	46 (53.5%)
Male	35 (40.7%)
Missing	5 (5.6%)
Race	
White	74 (86%)
Black or African American	3 (3.5%)
Missing	9 (10.5%)
Ethnicity	
Non‐Hispanic	63 (73.2%)
Hispanic	1 (1.2%)
Missing	22 (25.6%)
CFTR mutation	
Homozygous F508del	50 (58.1%)
Other	36 (41.9%)
Prior depression diagnosis	33 (38.4%)
Prior anxiety diagnosis	37 (43.0%)
On psychiatric medications	50 (58.1%)
	Mean ± SD
Age	33.0 ± 10.0
Pre E/T/I FEV_1_% predicted	59.4 ± 24.2
Pre E/T/I PHQ‐9	5.0 ± 5.8
Pre E/T/I GAD‐7	4.9 ± 5.3

### 3.2 Overall Change in Depression and Anxiety Symptom Scores and Impact of COVID‐19

**FIGURE 1:**
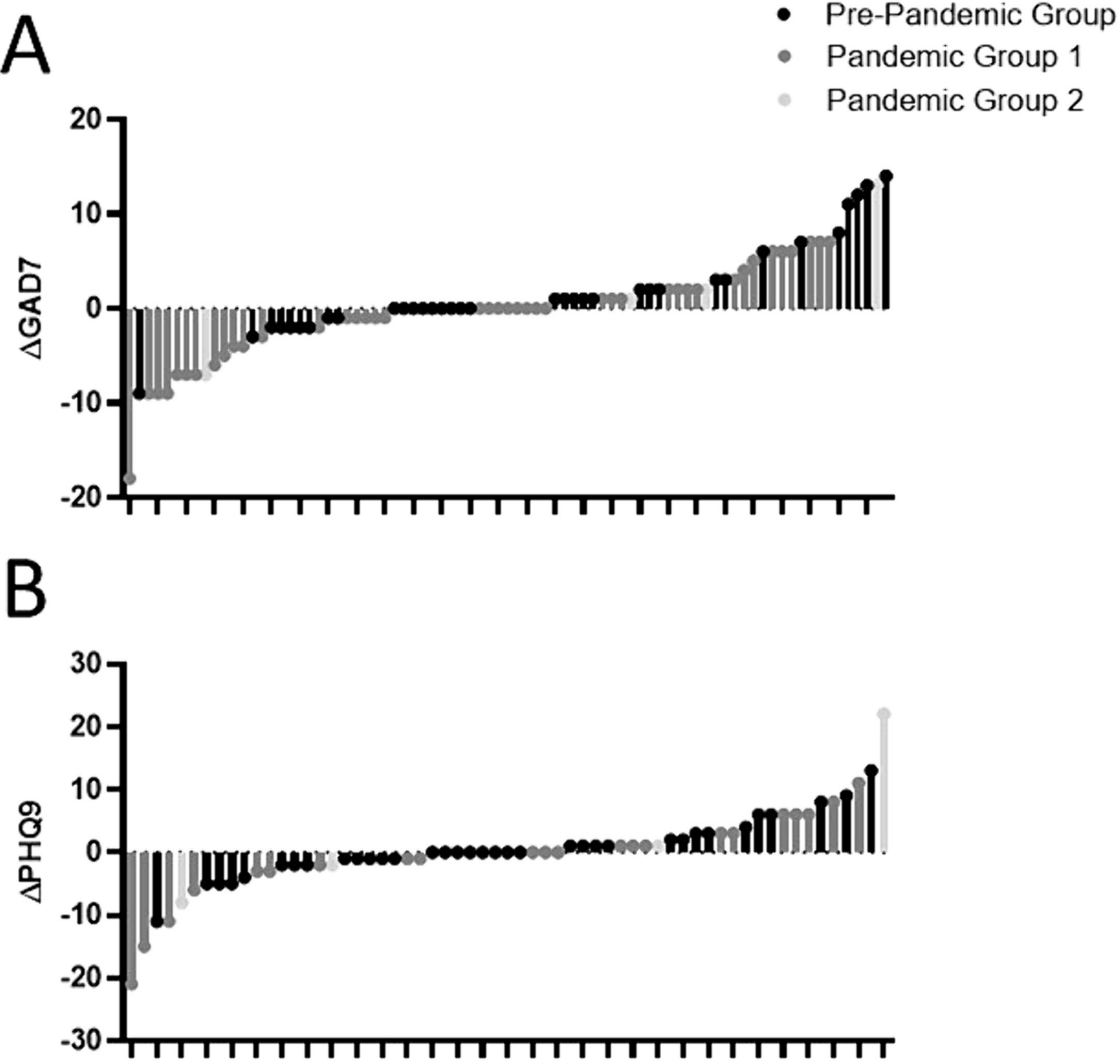
Waterfall plot of individual changes in GAD‐7 (upper) and PHQ‐9 (lower) after elexacaftor/tezacaftor/ivacaftor (E/T/I) according to relationship to the COVID‐19 pandemic. Groups are broken down by patients where all data collected pre‐pandemic = Pre‐Pandemic Group; Baseline data pre‐pandemic and post‐E/T/I data during pandemic = Pandemic Group 1; all data collected during pandemic = Pandemic Group 2.

Overall, there was no change in mean GAD‐7 (0.5 ± 5.3, *p* = 0.41) or PHQ‐9 (−0.02 ± 6.0, *p* = 0.97) scores following initiation of E/T/I. We examined the proportion of patients with a clinically significant change in their GAD‐7 score and found that roughly equal numbers of patients had clinically meaningful worsening [15 (18.5%)] and improvement in symptoms [11 (13.6%)] (Figure 1A). Similarly, 12 (15.2%) patients had a clinically meaningful worsening in PHQ‐9 and 11 (13.9%) had a clinically meaningful improvement (Figure 1B). In logistic regression analyses, age, gender, race, pre‐initiation FEV_1_pp, CFTR variant, and being prescribed psychiatric medication at baseline were not associated with experiencing a clinically significant worsening in GAD‐7 or PHQ‐9 post‐E/T/I.

Given the overlap between E/T/I approval and the onset of the COVID‐19 pandemic, we divided patients into 3 pandemic groups to assess pandemic effects on mental health symptom changes. While changes in PHQ‐9 score were not different between these three groups, we did observe a small but significant within‐group increase from baseline in GAD‐7 scores in the pre‐pandemic group (change 1.8 ± 4.9, *p* = 0.03) (Table S2), despite no change in the overall population or the other pandemic groups. There were no significant associations between age, gender, race, ethnicity, CFTR variant, FEV_1_pp, prescribed psychiatric medications at baseline, or prior diagnoses of anxiety or depression to explain the difference in change in GAD‐7 between the Pre‐Pandemic Group and Pandemic Group 1.

### 3.3 Effect of Historical Mental Health on Anxiety and Depression After E/T/I

As there may be a clinical diagnosis of anxiety or depression without active symptoms on GAD‐7/PHQ‐9, or conversely, a patient may report symptoms but not carry a clinical diagnosis, we separately evaluated the effects of baseline mental health on subsequent mental health after starting E/T/I.

#### 3.3.1 Prior Clinical Diagnosis of Anxiety

Thirty‐seven patients (43.0%) had a clinical diagnosis of anxiety in their medical record prior to E/T/I initiation. Baseline GAD‐7 score in this subgroup was 7.5 ± 5.6. Thirty‐five of these patients had pre‐ and post‐E/T/I GAD‐7 scores available. In this subgroup with a prior diagnosis of anxiety, numerically more patients experienced a clinically meaningful improvement in GAD‐7 score (*N* = 10/35) than a deterioration in GAD‐7 score (*N* = 6/35) (Table 2). Using logistic regression, a prior diagnosis of anxiety was not associated with increased odds of experiencing a clinically significant worsening in GAD‐7 score after E/T/I, versus those with no prior diagnosis of anxiety (OR = 0.85; 95% CI: 0.27, 2.66; *p* = 0.78).

**TABLE 2:** Anxiety and depression symptom change by subgroup.

	Clinical history of anxiety prior to ETI	No history of clinical Dx
Anxiety	Number (%)	Number (%)
Symptoms improved	10 (28.6)	1 (2.2)
Symptoms worsened	6 (17.1)	9 (19.6)
Symptoms unchanged	19 (54.3)	36 (78.3)
Total	35	46
	Clinical history of depression prior to ETI	No history of clinical Dx
Depression	Number (%)	Number (%)
Symptoms improved	6 (18.8)	5 (10.6)
Symptoms worsened	8 (25)	4 (8.5)
Symptoms unchanged	18 (56.3)	38 (80.9)
Total	32	47
	Prescribed psych meds at baseline	Not prescribed psych meds at baseline
Anxiety	Number (%)	Number (%)
Symptoms improved	11 (22.0)	0 (0.0)
Symptoms worsened	10 (20.0)	5 (16.1)
Symptoms unchanged	29 (58.0)	26 (83.9)
Total	50	31
	Prescribed psych meds at baseline	Not prescribed psych meds at baseline
Depression	Number (%)	Number (%)
Symptoms improved	10 (20.8)	1 (3.2)
Symptoms worsened	8 (16.7)	4 (12.9)
Symptoms unchanged	30 (62.5)	26 (83.9)
Total	48	31
	GAD‐7 ≥ 5 at pre‐initiation assessment	GAD‐7 < 5 at pre‐initiation assessment
Anxiety	Number (%)	Number (%)
Symptoms improved	11 (36.7)	0 (0)
Symptoms worsened	5 (16.7)	10 (19.6)
Symptoms unchanged	14 (46.7)	41 (80.4)
Total	30	51
	PHQ‐9 ≥ 5 at pre‐initiation assessment	PHQ‐9 < 5 at pre‐initiation assessment
Depression	Number (%)	Number (%)
Symptoms improved	11 (35.5)	0 (0)
Symptoms worsened	4 (12.9)	8 (16.7)
Symptoms unchanged	16 (51.6)	40 (83.3)
Total	31	48

#### 3.3.2 Anxiety Symptoms on Baseline GAD‐7 Score

Thirty patients (37%) had a GAD‐7 score ≥5 before starting E/T/I. Baseline GAD‐7 score in this subgroup was 10.7 ± 4.4 and twenty‐one (70%) of these patients had a prior clinical diagnosis of anxiety in their medical record. More patients in this subgroup experienced clinically meaningful improvement in GAD‐7 score (*N* = 11/30) than a deterioration in GAD‐7 score (*N* = 5/30) (Table 2). No other clinical factors (age, gender, FEV_1_pp, CFTR variant, prior diagnosis of depression, or baseline PHQ‐9 ≥ 5) were associated with clinically significant worsening of GAD‐7 score after E/T/I initiation.

#### 3.3.3 Prior Clinical Diagnosis of Depression

Thirty‐three (38.4%) patients had a diagnosis of depression in their medical record prior to E/T/I initiation. Baseline PHQ‐9 in this group was 8.0 ± 7.0. Thirty‐two of these patients had both pre‐ and post‐E/T/I PHQ‐9 scores available. An approximately equal fraction of these patients had a clinically meaningful improvement (6/32) or deterioration (8/32) in PHQ‐9 score after E/T/I (Table 2). However, a greater proportion of those with a prior diagnosis of depression experienced a clinically meaningful worsening in PHQ‐9 score (8/32) than those without a pre‐existent depression diagnosis (4/47). Using logistic regression, we determined that a prior diagnosis of depression approached statistical significance with an odds ratio of 3.58 (95%CI: 0.98, 13.13; *p* = 0.054) for experiencing a clinically significant worsening in PHQ‐9 score after E/T/I, versus those with no prior depression diagnosis.

#### 3.3.4 Depression Symptoms on Baseline PHQ‐9 Scores

In the total data set, 32 (39.0%) patients had elevated PHQ‐9 scores before E/T/I initiation (PHQ‐9 ≥ 5). Baseline PHQ‐9 score in this subgroup was 10.4 ± 5.8. Twenty (62.5%) of these patients had a prior clinical diagnosis of depression in their medical record. More of these patients with a high baseline PHQ‐9 score had a meaningful improvement in this score (N = 11/31) than a deterioration in PHQ‐9 score (*N* = 4/31) (Table 2). No other clinical factors we examined (age, gender, FEV_1_pp, CFTR variant, prior diagnosis of depression, or baseline GAD‐7 ≥ 5) were associated with clinically significant worsening of PHQ‐9 score after E/T/I initiation.

### 3.4 Effect of Being Prescribed Psychiatric Medications at Baseline on Anxiety and Depression After E/T/I

Fifty‐three patients were prescribed psychiatric medications at baseline (Supplementary Table 2). Of those, 31 (58.5%) had a diagnosis of depression and 31 (58.5%) had a diagnosis of anxiety pre‐E/T/I. The baseline GAD‐7 score in this group was 6.6 ± 5.8, and the baseline PHQ‐9 score was 6.4 ± 6.4. Pre‐ and post‐E/T/I GAD‐7 data were available for 50 patients. Approximately equal numbers of patients in this group experienced clinically meaningful improvement (11/50) and clinically meaningful worsening (10/50) of anxiety symptoms (Table 2). Forty‐eight patients prescribed psychiatric medications at baseline had pre‐ and post‐E/T/I PHQ‐9 data. In this group, similar numbers of individuals experienced clinically meaningful improvement of depression symptoms (10/48) and clinically meaningful worsening (8/48) of symptoms (Table 2). Being prescribed a psychiatric medication at baseline was not associated with worsening change in either GAD‐7 (*p* = 0.66) or PHQ‐9 (*p* = 0.65).

### 3.5 Psychiatric Medication Changes During the Observed Time Period

Eighteen patients (20.9%) had psychiatric medication added, increased, or underwent multiple adjustments during the 6 months post‐E/T/I (Supplementary Table 3). Of those patients, 11 (61.1%) had a diagnosis of both depression and anxiety, 4 (22.2%) had depression only, and 2 (11.1%) had a diagnosis of anxiety only pre‐E/T/I. Only 1 patient (5.6%) in that group had neither depression or anxiety pre‐E/T/I. Eight patients (9.3%) had psychiatric medications decreased or stopped. Of those patients, 5 (62.5%) had a diagnosis of depression and 4 (50%) had a diagnosis of anxiety pre‐E/T/I. Of the 33 patients who had a diagnosis of depression pre‐E/T/I, 15 (45.4%) had psychiatric medications added, increased, or underwent multiple adjustments post‐E/T/I, while 5 (15.1%) had medications decreased or stopped. Of the 37 patients who had a diagnosis of anxiety pre‐E/T/I, 13 (35.1%) had psychiatric medications added, increased, or underwent multiple adjustments post‐E/T/I, while 4 (10.8%) had medications decreased or stopped.

## 4 Discussion

In a retrospective cohort analysis of adult patients with CF, we found no significant overall changes in anxiety or depression symptoms (as measured by GAD‐7 and PHQ‐9 scores) after initiation of E/T/I. A clinically meaningful deterioration or improvement of anxiety or depression symptoms was seen in an equivalent number of patients. Having a prior diagnosis of depression showed a trend towards a greater likelihood of experiencing a significant worsening in PHQ‐9 score, but the result was not statistically significant. Having a prior diagnosis of anxiety did not appear to predict a higher risk of worsened mental health after initiating E/T/I. Similarly, other clinical factors (age, gender, lung function, being prescribed a psychiatric medication at baseline, etc.) were not associated with worsening in GAD‐7 or PHQ‐9 scores after E/T/I. For individuals who do not have depression or anxiety symptoms or prior diagnoses pre‐initiation, these data predict that the majority of patients (80%–90%) will not see worsening in depression and anxiety symptom scores post‐initiation of treatment. However, our data suggest that there may be a higher risk of worsened depression symptoms in those with a prior clinical diagnosis of depression, regardless of whether they have elevated depression symptoms at the time of E/T/I initiation. In contrast, a prior diagnosis of anxiety did not predict worsened mental health symptoms. In those with a history of depression, closer monitoring for worsened depression symptoms after E/T/I prescription should be considered.

Overall, our findings are consistent with prior studies which suggest no overall population effect on depression or anxiety symptoms with E/T/I initiation. The largest systematic review to date, incorporating data from multicenter clinical trials, sponsor‐held safety databases, post‐marketing surveillance, and existing scientific literature, found no increase in depression‐related events or suicidialty [29]. Notably, they reported similar prevalence of depression after E/T/I start compared with registry reports of 5 years of data collected before E/T/I initiation.

Our study adds to the existing literature by closely examining the effects of a prior clinical diagnosis of depression or anxiety, elevated baseline PHQ‐9 or GAD‐7, or psychotropic medication use at baseline to provide a more comprehensive picture of depression/anxiety in the context of E/T/I initiation. Furthermore, we attempted to control for any confounding effects presented by the COVID‐19 pandemic. Taken together, interpretation of these collective data is complicated due to multiple factors that likely contribute to the mental health of pwCF post‐E/T/I initiation [35]. Teasing out these factors at a population level to make clinical decisions about an individual may not be particularly helpful, though the issue overall is clearly concerning to the entire CF community. The Cystic Fibrosis Foundation Mental Health Advisory Committee recently surveyed 75 US CF physicians and reported physicians harbored concern over E/T/I's relationship with worsening stress, anxiety and depression [36]. Clinicians will likely need to understand which factors may play a role in both improvements and worsening of mental health symptoms after initiation of E/T/I in order to have an educated risk/benefit discussion with their patients, and our data help to further define the many ways that depression and anxiety diagnoses are manifested in clinical care, including patients with elevated screening scores and patients reporting symptoms but not having a clinical diagnosis. It may be beneficial to incorporate mental health professionals in these discussions, especially for individuals with depression diagnoses.

Around 20% of patients in our study had psychiatric medications increased, added, or underwent multiple changes post‐E/T/I, while only 9.3% had these medications decreased or discontinued. These data are similar to that of Zhang and colleagues [26]. Causality of psychiatric medication changes is difficult to discern in a retrospective chart review study. A medication class may be changed because of undesirable side effects, even if it is controlling the symptoms of anxiety or depression. A prospective analysis of depression and anxiety symptoms at the time of any psychiatric medication adjustments would be more meaningful.

We saw no overt effect of the COVID‐19 pandemic on our overall results. Interestingly, we observed a significant worsening of the GAD‐7 score only in the pre‐pandemic group. One possible explanation is that initiation of E/T/I may not have occurred randomly, with “early adopters” perhaps being more prone to worsened anxiety. Additionally, while we defined the pandemic onset as the date that the world declaration was made, there was ongoing consternation about the SARS‐CoV2 virus for several months ahead of this date, and these early uncertainties and worry may have contributed to worsened anxiety scores in the pre‐pandemic population. Overall, it is reassuring that our data did not show significant worsening in depression or anxiety symptoms in our study population during COVID‐19 and is in line with prior studies that suggest that pwCF did not see increases in psychological distress during the onset of the pandemic [33, 34, 35, 36].

Our study has several limitations that may affect interpretation of our results. First, our single center study collected data retrospectively on a small population of pwCF, and it is possible we are underpowered to see a significant effect. Next, we did not have a disease control group of scores in people who did not take E/T/I. Additionally, we were unable to assess any other mental health interventions in our study, such as ongoing work with a psychotherapist, which may also have impacted scores. However, our results generally align with anecdotal clinical experience, as well as with the findings of another recent retrospective study of mental health symptoms post‐E/T/I [25].As a retrospective study, we were unable to collect GAD‐7 or PHQ‐9 scores at standardized time points before and after initiation of E/T/I. In fact, our median time from drug initiation to post‐initiation score collection was over 6 months, and the baseline assessment was often >1 year before E/T/I initiation. Given the long duration until score collection post‐E/T/I, it is possible that patients discontinued E/T/I or reduced the dosage of the medication in the interval time period. This would have been missed in our data collection. Further, the nature of these clinically collected data prevents us from describing the onset or trajectory of mental health symptoms after E/T/I. A prospective study with questionnaire collection at set timepoints after drug initiation would provide stronger evidence about direct effects of E/T/I on depression or anxiety symptoms. Such a study that was recently published showed a small, but significant decrease in PHQ‐9 scores 8–16 weeks after E/T/I, but no change in GAD‐7 scores [27]. They also found that this difference was driven by a decrease in depressive symptoms in males, but not females in their cohort. However, they did not assess prior psychiatric diagnoses, psychotropic medication use, or account for any confounding effects of the COVID‐19 pandemic. Our study looks at symptom scores further out from initiation of therapy and associated with changes in medication.

An additional consideration is that we focused on symptoms associated with anxiety and depression as part of our study using commonly collected screening tools. We are unable to assess other potential neuropsychiatric effects of E/T/I, such as cognitive changes (“brain fog,” memory deficits, changes in processing speed, etc.), agitation, aggression, or hallucinations. More detailed clinical assessments would be needed to examine the possible extent of neuropsychiatric adverse effects of E/T/I [28]. Our patients had baseline higher rates of anxiety and depression than other cited studies, which may have impacted our results. Finally, our study did not include children or adolescents, and it is possible that effects may be different in this population. This is an important question, particularly with the completion of a phase 3 study of E/T/I in children age 2–5 [37] and subsequent FDA application to extend the approval age down to age 2 [22].

Overall, our study adds important data to the ongoing debate regarding a link between E/T/I treatment and symptoms of anxiety or depression. While there do not appear to be pervasive negative effects on mental health at the population level, it remains likely that there are individuals who are more susceptible to direct neurochemical effects of the medication that are not yet understood. A small percentage of patients experiencing significant deterioration in mental health may not lead to differences in population data, as we have described, but is still an important clinical concern for an individual. This work, combined with existing knowledge, can hopefully support medical and mental health clinicians and pwCF when making decisions about initiating E/T/I. Mental health clinicians working with pwCF should be aware of the potential need for more intensive symptom monitoring and treatment in certain individuals after initiation of E/T/I, especially those diagnosed with depression.

## Author Contributions

H.P. conceptualized the study, curated data, reviewed and edited the manuscript. M.Z.P. curated and validated data and reviewed and edited the manuscript. A.C. developed methodology, conducted formal analyses, validated data, and reviewed and edited the manuscript. S.H.D. conceptualized the study, provided supervision, and reviewed and edited the manuscript. J.L.G. conceptualized the study, helped with methodology development, supervised the study, and reviewed and edited the manuscript. NAS conceptualized the study, supervised H.P., wrote the original draft manuscript, and participated in reviewing and editing the final manuscript. All authors provided approval of the submitted manuscript.

## Ethics Statement

The study was approved by the Institutional Review Board, and informed consent was waived due to the use of retrospective and de‐identified data. The study adhered to the guidelines set forth by the Office of Human Research Protection that is supported by the US Department of Health and Human Services.

## Conflicts of Interest

The authors declare no conflicts of interest.

## Supporting information


**Table S1.** Psychiatric medications per patient prior to Elexacaftor/Tezacaftor/Ivacaftor initiation.
**Table S2.** Analysis of the effect of COVID‐19 on anxiety and depression symptoms post‐initiation of Elexacaftor/Tezacaftor/Ivacaftor.
**Table S3.** Patient Characteristics and Screener Scores based on Psychiatric Medication Classification in the 6 months post‐E/T/I.

## Data Availability

The data that support the findings of this study are available on request from the corresponding author. The data are not publicly available due to privacy or ethical restrictions.

## References

[1] B. Klimova , K. Kuca , M. Novotny , and P. Maresova , “Cystic Fibrosis Revisited ‐ A Review Study,” Medicinal Chemistry 13, no. 2 (2017): 102–109, 10.2174/1573406412666160608113235. 27292156

[2] S. M. Moskowitz , J. F. Chmiel , D. L. Sternen , E. Cheng , and G. R. Cutting , “CFTR‐Related Disorders,” in GeneReviews(®), eds. R. A. Pagon , M. P. Adam , H. H. Ardinger , et al. (Seattle: University of Washington, 1993).

[3] J. S. Talwalkar , J. L. Koff , H. B. Lee , C. J. Britto , A. M. Mulenos , and A. M. Georgiopoulos , “Cystic Fibrosis Transmembrane Regulator Modulators: Implications for the Management of Depression and Anxiety in Cystic Fibrosis,” Psychosomatics 58, no. 4 (2017): 343–354, 10.1016/j.psym.2017.04.001. 28576305

[4] A. L. Quittner , L. Goldbeck , J. Abbott , et al., “Prevalence of Depression and Anxiety in Patients With Cystic Fibrosis and Parent Caregivers: Results of the International Depression Epidemiological Study Across Nine Countries,” Thorax 69, no. 12 (2014): 1090–1097, 10.1136/thoraxjnl-2014-205983. 25246663

[5] M. M. Rafeeq and H. A. S. Murad , “Cystic Fibrosis: Current Therapeutic Targets and Future Approaches,” Journal of Translational Medicine 15, no. 1 (2017): 84, 10.1186/s12967-017-1193-9. 28449677 PMC5408469

[6] C. L. Ren , R. L. Morgan , C. Oermann , et al., “Cystic Fibrosis Foundation Pulmonary Guidelines. Use of Cystic Fibrosis Transmembrane Conductance Regulator Modulator Therapy in Patients With Cystic Fibrosis,” Annals of the American Thoracic Society 15, no. 3 (2018): 271–280, 10.1513/AnnalsATS.201707-539OT. 29342367

[7] D. Shiferaw and S. Faruqi , “Profile of Tezacaftor/Ivacaftor Combination and Its Potential in the Treatment of Cystic Fibrosis,” Therapeutics and Clinical Risk Management 15 (2019): 1029–1040, 10.2147/TCRM.S165027. 31692517 PMC6710479

[8] V. Garg , J. Shen , C. Li , et al., “Pharmacokinetic and Drug‐Drug Interaction Profiles of the Combination of Tezacaftor/Ivacaftor,” Clinical and Translational Science 12, no. 3 (2019): 267–275, 10.1111/cts.12610. 30694595 PMC6510372

[9] S. L. Paterson , P. J. Barry , and A. R. Horsley , “Tezacaftor and Ivacaftor for the Treatment of Cystic Fibrosis,” Expert Review of Respiratory Medicine 14, no. 1 (2020): 15–30, 10.1080/17476348.2020.1682998. 31626570

[10] J. L. Taylor‐Cousar , A. Munck , E. F. McKone , et al., “Tezacaftor‐Ivacaftor in Patients With Cystic Fibrosis Homozygous for Phe508del,” New England Journal of Medicine 377, no. 21 (2017): 2013–2023, 10.1056/NEJMoa1709846. 29099344

[11] S. M. Rowe , C. Daines , F. C. Ringshausen , et al., “Tezacaftor‐Ivacaftor in Residual‐Function Heterozygotes With Cystic Fibrosis,” New England Journal of Medicine 377, no. 21 (2017): 2024–2035, 10.1056/NEJMoa1709847. 29099333 PMC6472479

[12] T. Kirby , “Tezacaftor‐Ivacaftor Is Safe and Efficacious in Patients With Cystic Fibrosis With Phe508del Mutations,” Lancet Respiratory Medicine 6, no. 1 (2018): 13–14, 10.1016/S2213-2600(17)30439-3. 29248431

[13] S. T. Lommatzsch and J. L. Taylor‐Cousar , “The Combination of Tezacaftor and Ivacaftor in the Treatment of Patients With Cystic Fibrosis: Clinical Evidence and Future Prospects in Cystic Fibrosis Therapy,” Therapeutic Advances in Respiratory Disease 13 (2019): 1753466619844424, 10.1177/1753466619844424. 31027466 PMC6487765

[14] S. H. Donaldson , J. M. Pilewski , M. Griese , et al., “Tezacaftor/Ivacaftor in Subjects With Cystic Fibrosis and F508del/F508del‐CFTR or F508del/G551D‐CFTR,” American Journal of Respiratory and Critical Care Medicine 197, no. 2 (2018): 214–224, 10.1164/rccm.201704-0717OC. 28930490 PMC5768901

[15] Tezacaftor/Ivacaftor for Cystic Fibrosis,” Australian Prescriber 42, no. 5 (2019): 174–175, 10.18773/austprescr.2019.060. 31631935 PMC6787300

[16] Elexacaftor/Tezacaftor/Ivacaftor (Trikafta) for Cystic Fibrosis,” Medical Letter on Drugs and Therapeutics 62, no. 1589 (2020): 5–7. 31999662

[17] S. Walker , P. Flume , J. McNamara , et al., “A Phase 3 Study of Tezacaftor in Combination With Ivacaftor in Children Aged 6 Through 11 Years With Cystic Fibrosis,” Journal of Cystic Fibrosis 18, no. 5 (2019): 708–713, 10.1016/j.jcf.2019.06.009. 31253540

[18] H. G. M. Heijerman , E. F. McKone , D. G. Downey , et al., “Efficacy and Safety of the Elexacaftor Plus Tezacaftor Plus Ivacaftor Combination Regimen in People With Cystic Fibrosis Homozygous for the F508del Mutation: A Double‐Blind, Randomised, Phase 3 Trial,” Lancet 394, no. 10212 (2019): 1940–1948, 10.1016/S0140-6736(19)32597-8. 31679946 PMC7571408

[19] N. Kapouni , M. Moustaki , K. Douros , and I. Loukou , “Efficacy and Safety of Elexacaftor‐Tezacaftor‐Ivacaftor in the Treatment of Cystic Fibrosis: A Systematic Review,” Children (Basel) 10, no. 3 (2023): 554, 10.3390/children10030554. 36980112 PMC10047761

[20] C. E. Bear , “A Therapy for Most With Cystic Fibrosis,” Cell 180, no. 2 (2020): 211, 10.1016/j.cell.2019.12.032. 31978337

[21] V. Sergeev , F. Y. Chou , G. Y. Lam , C. M. Hamilton , P. G. Wilcox , and B. S. Quon , “The Extrapulmonary Effects of Cystic Fibrosis Transmembrane Conductance Regulator Modulators in Cystic Fibrosis,” Annals of the American Thoracic Society 17, no. 2 (2020): 147–154, 10.1513/AnnalsATS.201909-671CME. 31661636 PMC6993798

[22] C. J. McKinzie , J. L. Goralski , T. L. Noah , G. Z. Retsch‐Bogart , and M. B. Prieur , “Worsening Anxiety and Depression After Initiation of Lumacaftor/Ivacaftor Combination Therapy in Adolescent Females With Cystic Fibrosis,” Journal of Cystic Fibrosis 16, no. 4 (2017): 525–527, 10.1016/j.jcf.2017.05.008. 28602538

[23] S. Heo , D. C. Young , J. Safirstein , et al., “Mental Status Changes During Elexacaftor/Tezacaftor/Ivacaftor Therapy,” Journal of Cystic Fibrosis 21, no. 2 (2022): 339–343, 10.1016/j.jcf.2021.10.002. 34742667

[24] G. Spoletini , L. Gillgrass , K. Pollard , et al., “Dose Adjustments of Elexacaftor/Tezacaftor/Ivacaftor in Response to Mental Health Side Effects in Adults With Cystic Fibrosis,” Journal of Cystic Fibrosis 15 (2022): 1061–1065, 10.1016/j.jcf.2022.05.001. 35585012

[25] L. Zhang , D. Albon , M. Jones , and H. Bruschwein , “Impact of Elexacaftor/Tezacaftor/Ivacaftor on Depression and Anxiety in Cystic Fibrosis,” Therapeutic Advances in Respiratory Disease 16 (2022): 17534666221144212, 10.1177/17534666221144211. PMC9793010 36562554

[26] H. Pham , M. Vandeleur , R. M. Mainzer , and S. Ranganathan , “Mental Health, Sleep, and Respiratory Health After Initiating Elexacaftor/Tezacaftor/Ivacaftor Treatment in Children With Cystic Fibrosis,” Pediatric Pulmonology (2024): 1–8, 10.1002/ppul.27100. 38860602

[27] L. Piehler , R. Thalemann , C. Lehmann , et al., “Effects of Elexacaftor/Tezacaftor/Ivacaftor Therapy on Mental Health of Patients With Cystic Fibrosis,” Frontiers in Pharmacology 14 (2023): 1179208, 10.3389/fphar.2023.1179208. 37153809 PMC10160464

[28] S. Graziano , F. Boldrini , G. R. Pellicano , et al., “Longitudinal Effects of Elexacaftor/Tezacaftor/Ivacaftor: Multidimensional Assessment of Neuropsychological Side Effects and Physical and Mental Health Outcomes in Adolescents and Adults,” Chest 165, no. 4 (2024): 800–809, 10.1016/j.chest.2023.10.043. 37925143

[29] B. Ramsey , C. U. Correll , D. R. DeMaso , et al., “Elexacaftor/Tezacaftor/Ivacaftor Treatment and Depression‐Related Events,” American Journal of Respiratory and Critical Care Medicine 209, no. 3 (2024): 299–306, 10.1164/rccm.202308-1525OC. 37890129 PMC10840763

[30] H. Pudukodu , K. Howe , S. Donaldson , et al., “281: Worsening Anxiety After Initiation of Elexacaftor/Tezacaftor/Ivacaftor in an Adult Cohort of Patients With Cystic Fibrosis,” Journal of Cystic Fibrosis 20 (2021): S135–S136, 10.1016/S1569-1993(21)01706-9.

[31] K. Kroenke , R. L. Spitzer , J. B. W. Williams , and B. Löwe , “The Patient Health Questionnaire Somatic, Anxiety, and Depressive Symptom Scales: A Systematic Review,” General Hospital Psychiatry 32, no. 4 (2010): 345–359, 10.1016/j.genhosppsych.2010.03.006. 20633738

[32] R. L. Spitzer , K. Kroenke , J. B. W. Williams , and B. Löwe , “A Brief Measure for Assessing Generalized Anxiety Disorder: The GAD‐7,” Archives of Internal Medicine 166, no. 10 (2006): 1092–1097, 10.1001/archinte.166.10.1092. 16717171

[33] P. A. Harris , R. Taylor , R. Thielke , J. Payne , N. Gonzalez , and J. G. Conde , “Research Electronic Data Capture (REDCap): A Metadata‐Driven Methodology and Workflow Process for Providing Translational Research Informatics Support,” Journal of Biomedical Informatics 42, no. 2 (2009): 377–381, 10.1016/j.jbi.2008.08.010. 18929686 PMC2700030

[34] “Coronavirus Disease (COVID‐19) Pandemic,” accessed June 28, 2024, https://www.who.int/europe/emergencies/situations/covid‐19.

[35] M. B. VanElzakker , E. M. Tillman , L. M. Yonker , E.‐M. Ratai , and A. M. Georgiopoulos , “Neuropsychiatric Adverse Effects From CFTR Modulators Deserve a Serious Research Effort,” Current Opinion in Pulmonary Medicine 29, no. 6 (2023): 603–609, 10.1097/MCP.0000000000001014. 37655981 PMC10552811

[36] C. J. Bathgate , E. Muther , A. M. Georgiopoulos , et al., “Positive and Negative Impacts of Elexacaftor/Tezacaftor/Ivacaftor: Healthcare Providers' Observations Across US Centers,” Pediatric Pulmonology 58, no. 9 (2023): 2469–2477, 10.1002/ppul.26527. 37265418

[37] J. L. Goralski , J. E. Hoppe , M. A. Mall , et al., “Phase 3 Open‐Label Clinical Trial of Elexacaftor/Tezacaftor/Ivacaftor in Children Aged 2‐5 Years With Cystic Fibrosis and at Least one F508del Allele,” American Journal of Respiratory and Critical Care Medicine 208, no. 1 (2023): 59–67, 10.1164/rccm.202301-0084OC. 36921081 PMC10870849

