# Supplementary material for: Analysis of Depression and Anxiety Scores Following Initiation of Elexacaftor/Tezacaftor/Ivacaftor in Adults With Cystic Fibrosis
Source: Clin Respir J. 2024 Aug 29;18(9):e70007. doi: 10.1111/crj.70007 (PMC11362501; doi:10.1111/crj.70007)
Supplement: Supplementary file 1 — Table S1. Psychiatric medications per patient prior to Elexacaftor/Tezacaftor/Ivacaftor initiation. Table S2. Analysis of the effect of COVID‐19 on anxiety and depression symptoms post‐initiation of Elexacaftor/Tezacaftor/Ivacaftor. Table S3. Patient Characteristics and Screener Scores based on Psychiatric Medication Classification in the 6 months post‐E/T/I. [file CRJ-18-e70007-s001.docx]

Supplementary Table 1. Psychiatric medications per patient prior to Elexacaftor/Tezacaftor/Ivacaftor initiation

| **Medications** |
| --- |
| Alprazolam, Bupropion, Duloxetine, Gabapentin, Mirtazapine, Sertraline, Zolpidem |
| Alprazolam, Citalopram |
| Aripiprazole, Escitalopram, Zolpidem |
| Aripiprazole, Gabapentin, Venlafaxine, Vilazodone |
| Bupropion |
| Bupropion, Clonazepam, Dexmethylphenidate, Quetiapine |
| Bupropion, Hydroxyzine, Sertraline, Trazodone |
| Bupropion, Mirtazapine, Trazodone |
| Bupropion, Sertraline, Trazodone |
| Buspirone, Duloxetine, Fluoxetine, Sertraline |
| Buspirone, Vilazodone |
| Citalopram |
| Citalopram |
| Citalopram |
| Citalopram, Clonazepam, Duloxetine, Mirtazapine, Sertraline |
| Citalopram, Trazodone |
| Clonazepam |
| Clonazepam, Duloxetine, Paroxetine, Sertraline |
| Duloxetine, Fluoxetine, Sertraline |
| Escitalopram |
| Escitalopram |
| Escitalopram, Fluoxetine, Mirtazapine, Sertraline, Trazodone, Venlafaxine |
| Escitalopram, Mirtazapine |
| Escitalopram, Trazodone |
| Fluoxetine |
| Fluoxetine, Sertraline |
| Fluoxetine, Trazodone |
| Gabapentin, Sertraline |
| Hydroxyzine |
| Hydroxyzine |
| Hydroxyzine |
| Lamotrigine |
| Lamotrigine, Quetiapine |
| Lorazepam |
| Mirtazapine |
| Mirtazapine |
| Mirtazapine |
| Mirtazapine |
| Mirtazapine |
| Mirtazapine |
| Mirtazapine, Sertraline |
| Sertraline |
| Sertraline |
| Sertraline |
| Sertraline |
| Sertraline |
| Sertraline |
| Sertraline |
| Sertraline |
| Sertraline, Trazodone |
| Trazodone |
| Zolpidem |

Supplementary Table 2. Analysis of the effect of COVID-19 on anxiety and depression symptoms post-initiation of Elexacaftor/Tezacaftor/Ivacaftor

|  | **Pre-Pandemic Group** | | **Pandemic Group 1** | | **Pandemic Group 2** | | **All** | |
| --- | --- | --- | --- | --- | --- | --- | --- | --- |
|  | **N** | **Mean ± STD** | **N** | **Mean ± STD** | **N** | **Mean ± STD** | **N** | **Mean ± STD** |
| Pre E/T/I GAD-7 | 35 | 3.9 ± 4.7 | 42 | 5.3 ± 5.7 | 4 | 9.5 ± 4.4 | 81 | 4.9 ± 5.3 |
| Post-E/T/I GAD-7 | 36 | 5.8 ± 6.3 | 46 | 4.3 ± 4.6 | 4 | 11.8 ± 5.4 | 86 | 5.3 ± 5.6 |
| ΔGAD-7 | 35 | **1.8 ± 4.9*** | 42 | -0.8 ± 5.2 | 4 | 2.3 ± 8.2 | 81 | 0.5 ± 5.3 |
| Pre E/T/I PHQ-9 | 36 | 4.1 ± 4.7 | 42 | 5.5 ± 6.3 | 4 | 7.8 ± 8.2 | 82 | 5 ± 5.8 |
| Post E/T/I PHQ-9 | 35 | 4.5 ± 4.7 | 44 | 4.5 ± 5.4 | 4 | 11 ± 11 | 83 | 4.8 ± 5.6 |
| ΔPHQ-9 | 35 | 0.5 ± 4.4 | 40 | -0.9 ± 6.3 | 4 | 3.3 ± 13 | 79 | 0 ± 6 |

E/T/I = Elexacaftor/Tezacaftor/Ivacaftor

Pandemic Group 1 = pre-E/T/I scores collected pre-pandemic; post-E/T/I scores collected during pandemic

Pandemic Group 2 = pre- and post-E/T/I scores collected during pandemic

*p = 0.03

Supplementary Table 3: Patient Characteristics and Screener Scores based on Psychiatric Medication Classification in the 6 months post-E/T/I.

|  | **Added Medication** | **Decreased or stopped medication** | **Increase Dosage** | **Multiple Changes** | **No medication change** | **Not prescribed medication** |
| --- | --- | --- | --- | --- | --- | --- |
| ***N*** | *5* | *8* | *4* | *9* | *28* | *32* |
| **Age** | 32.8 | 33.0 | 32.0 | 28.8 | 36.1 | 31.8 |
| *Gender* |  |  |  |  |  |  |
| **Female** | 3 | 2 | 3 | 6 | 18 | 14 |
| **Male** | 2 | 6 | 1 | 2 | 9 | 15 |
| *Race* |  |  |  |  |  |  |
| **White** | 5 | 8 | 3 | 8 | 25 | 25 |
| **Black or Af American** | 0 | 0 | 1 | 0 | 1 | 1 |
| **Depression** | 3 | 5 | 4 | 8 | 11 | 2 |
| **Anxiety** | 5 | 4 | 3 | 5 | 14 | 6 |
